# Preoperative serum h-FABP concentration is associated with postoperative incidence of acute kidney injury in patients undergoing cardiac surgery

**DOI:** 10.1186/1471-2261-14-117

**Published:** 2014-09-12

**Authors:** Mehmet Oezkur, Armin Gorski, Jennifer Peltz, Martin Wagner, Maria Lazariotou, Christoph Schimmer, Peter U Heuschmann, Rainer G Leyh

**Affiliations:** Department of Cardiovascular Surgery, University Hospital Würzburg, Würzburg, Germany; Institute of Clinical Epidemiology and Biometry, Comprehensive Heart Failure Center, University of Würzburg, Würzburg, Germany; Clinical Trial Center Würzburg, University Hospital Würzburg, Würzburg, Germany

## Abstract

**Background:**

Fatty acid binding protein (FABP) is an intracellular transport protein associated with myocardial damage size in patients undergoing cardiac surgery. Furthermore, elevated FABP serum concentrations are related to a number of common comorbidities, such as heart failure, chronic kidney disease, diabetes mellitus, and metabolic syndrome, which represent important risk factors for postoperative acute kidney injury (AKI). Data are lacking on the association between preoperative FABP serum level and postoperative incidence of AKI.

**Methods:**

This prospective cohort study investigated the association between preoperative h-FABP serum concentrations and postoperative incidence of AKI, hospitalization time and length of ICU treatment. Blood samples were collected according to a predefined schedule. The AKI Network definition of AKI was used as primary endpoint. All associations were analysed using descriptive and univariate analyses.

**Results:**

Between 05/2009 and 09/2009, 70 patients undergoing cardiac surgery were investigated. AKI was observed in 45 patients (64%). Preoperative median (IQR) h-FABP differed between the AKI group (2.9 [1.7–4.1] ng/ml) and patients without AKI (1.7 [1.1–3.3] ng/ml; p = 0.04), respectively. Patients with AKI were significantly older. No statistically significant differences were found for gender, type of surgery, operation duration, CPB-, or X-Clamp time, preoperative cardiac enzymes, HbA1c, or CRP between the two groups. Preoperative h-FABP was also correlated with the length of ICU stay (r_s_ = 0.32, p = 0.007).

**Conclusions:**

We found a correlation between preoperative serum h-FABP and the postoperative incidence of AKI. Our results suggest a potential role for h-FABP as a biomarker for AKI in cardiac surgery.

## Background

Fatty Acid Binding Protein (FABP) is an intracellular transport protein responsible for transporting long chained fatty acids to their destination and has organ specific subtypes (e.g. heart-type, liver-type and brain-type FABP) [1, 2]. Heart-type FABP (h-FABP) is a marker for myocardial ischemia and is associated with ECG changes, arrhythmia and mortality after cardiac surgery [3–8]. Furthermore, h-FABP is elevated in patients with heart insufficiency, diabetes mellitus, chronic kidney disease, metabolic syndrome and is positively correlated with age [1, 4, 9–11]. These entities also represent important risk factors for postoperative acute kidney injury (AKI), which is a common postoperative complication with an incidence of 20 to 40% after cardiac surgery with high impact on postoperative mortality [12–20].

Utilizing h-FABP as a single marker for the described variety of risk factors of AKI seems to be possible. But studies regarding the association of h-FABP’s pre-operative serum concentration and post-operative outcomes for patients undergoing operations with cardiopulmonary bypass (CPB) are lacking. Although postoperative l-FABP has been described as a marker for AKI, pre-operative h-FABP has not been investigated as an AKI marker in a cohort of cardiac surgical patients [14, 15, 17, 21–24].

In the current study, we aimed to explore the association between preoperative h-FABP levels and the occurrence of AKI in patients undergoing cardiac surgery within the setting of a prospective mono-center cohort study.

## Methods

The study protocol was approved by the Ethics Committee at the University Hospital of Würzburg. Adult (>18 years) patients scheduled for elective CABG surgery or single valve repair/replacement in the Department of Cardio-Thoracic Surgery at the University Hospital Würzburg, Germany were eligible if they provided written informed consent. Exclusion criteria comprised chronic kidney disease stage 3 or greater (i.e. eGFR_CKD-EPI_ <30 ml/min/1.73 m^2^), off pump surgery, acute inflammation (defined by C-reactive protein (CRP) > 4 mg/dl, and procalcitonin (PCT) > 1.0 μg/l), history of immunological or musculoskeletal disorders, stroke within 30 day prior to admission, preoperative resuscitation, preoperative electrical cardioversion, salvage or urgent operations (defined as preoperative beginning of cardiopulmonary support). All patients were operated with blood cardioplegia at 4°C applied every 20 minutes and in mild hypothermic perfusion (32°C).

Primary endpoint was defined as incidence of AKI according to acute kidney injury network (AKIN) (increase of serum creatinine by ≥0.3 within 48 hours), while baseline was set at admission to hospital, which was usually the day before operation; secondary endpoints were mechanical ventilation time, length of ICU stay, and hospitalization time [16].

Standard demographic data of patients was collected prospectively including sex, age, weight, height and waist circumference at the time of operation. Intraoperative data such as operation time, paracorporal bypass time and aortic cross clamping time were noted. Blood samples were taken at hospital admission, after induction of anaesthesia 30 minutes, 6, 24 and 48 hours after aortic cross clamping. Length of mechanical ventilation, length of ICU stay and hospitalization time was listed.

h-FABP was measured by ELISA (HK402 HUMAN H-FABP ELISE KIT, Hycult biotech, Netherlands) in ng/ml in our own laboratory from blood samples taken after introduction of anaesthesia and 30 minutes after aortic clamping. Creatinine was measured in central laboratory unit of the University of Wurzburg.

### Statistical analysis

Continuous variables are presented as mean and standard deviations or as medians and interquartile ranges. Categorical variables are presented in percentages. Normal distribution testing was performed with a Kolmogorov-Smirnov-Test. To test for differences across all groups, *χ*^2^-test, Fisher’s exact test, *t*-test, Mann–Whitney-*U*-Test were used. Correlation analyses were performed employing Spearman correlation. In logistic regression analysis modelling we investigated h-FABP, age and eGFR regarding their association with the risk for AKI. To assure normal distribution of h-FABP natural log was used. Two-sided p-values of ≤0.05 were considered as statistically significant. Analyses were performed with SPSS Version 21.

## Results

A total of 238 consecutive patients admitted to the department of cardiovascular surgery of the University of Würzburg between 05/2009 and 09/2009 were screened for participation in the study. Of those, 122 patients met the inclusion criteria and 70 were included in the study. Reasons for exclusion were: urgent surgery (32 patients), chronic kidney disease stage 3 or greater (14 patients), off pump surgery (5 patients), acute inflammation (1 patient).

Patient characteristics at baseline are shown in Table 1. Patients were predominantly male (80%) with a mean age of 67(±10) yrs. The mean preoperative eGFR was 88(±11) ml/min/1.73 m^2^, 69% (n = 48) of the cohort received CABG surgery, while 22 (31%) underwent single valve replacement. Preoperative median troponin T was 0.01 [IQR: 0.01–0.01].

**Table 1:** **Preoperative characteristics**

Variable	All patients (n = 70)	AKI (n = 45)	No AKI (n = 25)	P-value
h-FABP [ng/dl]	2.9 (1.4–3.9)	2.9 (1.7–4.1)	1.7 (1.1–3.3)	0.039
Age (yr)	67 (±10)	69 (±9)	64 (±11)	0.016
Male	56 (80%)	37 (82%)	19 (76%)	0.76
CABG	48 (69%)	33 (73%)	15 (60%)	0.29
Preoperative creatinine [mg/dl]	1.0 (±0.3)	1.0 (±0.2)	0.9 (±0.1)	0.50
eGFR–CKDEPI preoperative [ml/min/1,73 m2]	88 (±11)	87 (±10)	91 (±12)	0.17
Troponin T (mg/dl)	0.01 (0.01–0.01)	0.01 (0.01–0.01)	0.01 (0.01–0.01)	0.28
Waist Circumferrence [cm]	104 (±13)	102 (±13)	104 (±13)	0.86
Operation duration [min]	240 (±60)	244 (±60)	239 (±59)	0.91
CPB duration [min]	113 (±36)	113 (±38)	114 (±34)	0.41
X-Clamp duration [min]	82 (±30)	81 (±29)	85 (±30)	0.90
HbA1c [%]	6.0 (5.6–6.6)	5.9 (5.6–6.4)	6.0 (5.7–7.2)	1.0
CK [mg/dl]	80 (55–116)	80 (60–118)	100 (57–122)	0.97
Myoglobin [mg/dl]	44 (31–54)	46 (32–59)	37 (29–52)	0.15
C-reactive protein [mg/dl]	0.3 (0.1–0.6)	0.3 (0.1–0.6)	0.2 (0.1–0.3)	0.33

Data are mean (±SD), number (%) or median (IQR). CABG: coronary artery bypass grafting, CPB: cardio pulmonary bypass, X-Clamp: Aortic clamping.

A total of 45 patients (64.3%) achieved the primary endpoint of AKI, while only three patients experienced more severe stages of AKI, including the need for dialysis (4.3%).

Postoperative ischemia markers did not differ significantly between the two groups of AKI vs. non-AKI (see Table 2). Patients with AKI had to remain longer on the ICU, however, mechanical ventilation time and overall hospital stay did not differ (Table 3). Preoperative median (IQR) h-FABP differed between the AKI group (2.9 [1.7–4.1] ng/m) and patients without AKI (1.7 [1.1–3.3] ng/ml; p = 0.04), respectively. Preoperative h-FABP was also correlated with the length of ICU stay (r_s_ = 0.32, p = 0.007) and with hospitalization time (r_s_ = 0.308; p = 0.009). No relation was found for age with the length of ICU stay (r_s_ = 0.089, p = 0.47). h-FABP was associated with preoperative creatinine (r_s_ = 0.404, p = 0.001), eGFR (r_s_ = -0.603, p < 0.001), and myoglobin levels (r_s_ = 0.610, p < 0.001).

**Table 2:** **Postoperative results**

Variable	All patients (n = 70)	AKI (n = 45)	No AKI (n = 25)	P-value
h-FABP [ng/dl]	9.9 (9.5–10.1)	9.9 (9.7–10.4)	9.9 (9.2–10.1)	0.79
Creatinine Peak Within 48 h [mg/dl]	1.4 (±0.6)	1.7 (±0.6)	1.0 (±0.2)	<0.001
C-reactive protein [mg/dl]	0.2 (0.1–0.5)	0.2 (0.1–0.4)	0.1 (0.1–0.2)	0.54
CK [mg/dl]	230 (145–375)	244 (175–438)	254 (138–422)	0.87
Myoglobin [mg/dl]	261 (182–410)	300 (212–424)	210 (137–446)	0.38
Troponin T [μg/l]	0.3 (0.1–0.5)	0.4 (0.2–0.7)	0.2 (0.1–0.5)	0.29

Data are mean (±SD), number (%) or median (IQR). CABG: coronary artery bypass grafting, CPB: cardio pulmonary bypass, X-Clamp: Aortic clamping.

**Table 3:** **Ventilation time, ICU stay and hospitalization time**

Variable	All patients (n = 70)	AKI (n = 45)	No AKI (n = 25)	P-value
Hospitalization [d]	12 (11–15)	13 (11–15)	12 (10–15)	0.35
ICU Stay [h]	44 (21–83)	46 (22–92)	22 (18–47)	0.030
Ventilation Time [h]	16 (12–20)	15 (11–20)	15 (12–21)	0.49

Data are mean (±SD), number (%) or median (IQR). CABG: coronary artery bypass grafting, CPB: cardio pulmonary bypass, X-Clamp: Aortic clamping.

Although a number of preoperative variables (e.g., age, waist circumference, HbA1c, operation duration, x-clamp time, all p < 0.05) differed between CABG and valve patients, there was no statistically significant difference in the incidence of the primary endpoint AKI (p = 0.29). In logistic regression analysis, the association of elevated h-FABP levels with an increased risk for AKI diminished after adjustment for age and preoperative kidney function (Table 4). However, in the final elimination model only h-FABP was associated with the incidence of AKI independent from age and preoperative eGFR.

**Table 4:** **Univariate and multivariate models**

Variable	Univariate	Model I	Model II	Model III	Model IV	Final model (p < 0.1) OR (95% CI)
Ln(h-FABP)	2.29 (1.08–4.88)	2.29 (1.08–4.89)	1.82 (0.78–4.24)	2.24 (1.01–4.94)	1.79 (0.73–4.39)	2.29 (1.08–4.88)
eGFR	0.99 (0.97–1.02)	0.99 (0.97–1.02)	-	-	0.99 (0.96–1.02)	-
Age	1.06 (1.00–1.11)	-	1.04 (0.98–1.10)	-	1.03 (0.97–1.09)	-
Ln(TropT)	1.33 (0.74–2.39)	-	-	1.11 (0.60–2.05)	1.15 (0.62–2.13)	

Determinants of AKI; data is presented as OR (CI95%); ln(h-FABP) to achieve normal distribution ln of FABP was calculated; final model is based on backward elimination.

7 (10%) of the patients had an positive Troponin T (>0.05 mg/dl). Neither in uni- nor in multivariate analyses preoperative troponin T showed a statically significant association with the incidence of AKI (p > 0.05).

## Discussion

In our study preoperative h-FABP serum concentration was associated with the incidence of postoperative AKI as well as with a longer stay on the ICU.

Previous studies have shown that postoperative h-FABP elevation was a marker for intraoperative myocardial damage with postoperative ECG changings and elevation of established ischemia markers [5, 7]. In a similar setting, serum and plasma levels of h-FABP without an ischemic incident were found to be associated with poor outcome in end-stage heart failure patients with circulatory support [25]. The pathophysiology for the poorer outcome was explained by the myocardial damage indicated by h-FABP elevation. None of the studies investigated a possible association between preoperative h-FABP concentrations with postoperative complications, although h-FABP has been linked to preoperative risk factors like heart failure, metabolic syndrome, chronic kidney disease or diabetes mellitus [1, 2, 9, 11, 26]. None of the cited studies have approached our hypothesis.

The incidence of AKI is higher in our cohort than in recent literature. This might be due to the rigorous definitions of AKIN with only a slight creatinine increase of 0.3 mg/dl. In particular as our study was performed during the summer months, the preoperative fastening status thus potentially causing hypovolemia might have sensitised the kidney for intraoperative stress and thus the incidence of AKI. It was reassuring though that only few patients experienced more severe stages of AKI including dialysis as we also did not observe any perioperative mortality.

However, postoperative urinary l-FABP has been shown in several recent studies as a promising marker for the extent of AKI [21–24]. Preoperative levels of urinary or serum levels of l-FABP have not been reported to be associated with AKI yet.

### h-FABP as a marker for comorbidities

Patients with higher h-FABP values were older, had higher creatinine values and thus lower eGFR in the present study. Age and preoperative h-FABP were related to postoperative AKI. These findings were similar to the results in previous studies. A possible explanation is the association of h-FABP with a wide spectrum of comorbidities evident in patients undergoing cardiac surgery. DM and metabolic syndrome, which have been described as risk factors for AKI after CABG, were known to cause elevated h-FABP levels [11, 18, 27–31]. We did not screen for all symptoms of metabolic syndrome, but for waist circumference, and HbA1c. Neither waist circumference nor HbA1c were associated with AKI or with elevated preoperative h-FABP. But in CABG patients HbA1c and waist circumference were higher than in patients with valve surgery. In a larger cohort of CABG patients an association between metabolic syndrome, h-FABP and AKI might be detected.

Older age indicates increased risk for AKI after cardiac surgery. Furthermore, elderly patients tend to have higher h-FABP levels [1, 2, 18–20, 30]. Both effects could be observed in our results.

### Length of ICU stay

The second finding in the present study was the association between preoperative h-FABP and longer postoperative ICU treatment. A reasonable explanation for this phenomenon was the incidence of AKI, which was associated with a longer ICU stay itself. AKI and renal failure are known factors with an impact on ICU and hospitalization time in cardiac surgical patients [14]. By being associated with the incidence of AKI, h-FABP was also related to the length of ICU stay. Longer ICU stay is associated with higher costs and ICU capacity frequently is a limiting factor for cardiac surgery departments. Predicting possible lengths of stay for risk patients is of crucial importance for the cost-effectiveness and for decent organisation of departments. Therefore predictive markers need to be identified in further studies.

### Postoperative h-FABP

Postoperative h-FABP was not associated with AKI in the current study. h-FABP is a proven marker for myocardial damage and is known to be elevated after cardiac surgery. Postoperative h-FABP correlated with the established myocardial damage markers (myoglobin, troponin T and CK/CK-MB) in this study. None of the postoperative markers had an association with AKI, however, we might have missed true associations due to limited sample size and thus limited statistical power.

The presented study with a small number of patients was designed to create hypotheses for future investigations and has several limitations. A larger cohort is needed for multivariate analyses, e.g. to adjust for the effect of age and to investigate the different AKI stages in more detail to also further explore the association with the length of stay on the ICU. The screening for metabolic syndrome was incomplete, as well as was the information on further pathomechanisms for the incidence of AKI, such as red cell count, left ventricular ejection fraction, and hemodynamic instabilities. However, this information is lacking in literature as well. Other subtypes of FABP (liver, brain) might also interfere with h-FABP and should be measured in future studies. If the rather strict definition of AKI by the KDIGO can be transferred unmodified to cardiac surgical patients needs to be discussed as well.

## Conclusions

The findings of our study suggest an association between preoperative serum h-FABP levels and postoperative incidence of AKI and the length of ICU stay. The results justify further research of FABP as a possible predictor for adverse events after cardiac surgery within the setting of prospective studies requiring larger sample sizes.

## Abbreviations

AKI: Acute kidney injury
CABG: Coronary artery bypass grafting
CPB: Cardio pulmonary bypass
DM: Diabetes mellitus
ECG: Electrocardiography
FABP: Fatty acid binding protein
h-FABP: Heart-type fatty acid binding protein
ICU: Intensive care unit
l-FABP: Liver-type fatty acid binding protein
IQR: Interquartile range
LV-EF: Left ventricular ejection fraction
SD: Standard deviation.

## References

[1] Azzazy HM, Pelsers MM, Christenson RH (2006). Unbound free fatty acids and heart-type fatty acid-binding protein: diagnostic assays and clinical applications. Clin Chem.

[2] Glatz JF, van der Vusse GJ (1996). Cellular fatty acid-binding proteins: their function and physiological significance. Prog Lipid Res.

[3] Fransen EJ, Maessen JG, Hermens WT, Glatz JF, Buurman WA (2000). Peri-operative myocardial tissue injury and the release of inflammatory mediators in coronary artery bypass graft patients. Cardiovasc Res.

[4] Hayashida N, Chihara S, Akasu K, Oda T, Tayama E, Kai E, Kawara T, Aoyagi S (2000). Plasma and urinary levels of heart fatty acid-binding protein in patients undergoing cardiac surgery. Jpn Circ J.

[5] Petzold T, Feindt P, Sunderdiek U, Boeken U, Fischer Y, Gams E (2001). Heart-type fatty acid binding protein (hFABP) in the diagnosis of myocardial damage in coronary artery bypass grafting. Eur J Cardiothorac Surg.

[6] Nakata T, Hashimoto A, Hase M, Tsuchihashi K, Shimamoto K (2003). Human heart-type fatty acid-binding protein as an early diagnostic and prognostic marker in acute coronary syndrome. Cardiology.

[7] Liu H, Dong GH, Xu B, Shen Y, Jing H (2005). Heart fatty acid binding protein in the rapid evaluation of myocardial damage following valve replacement surgery. Clin Chim Acta.

[8] Colli A, Josa M, Pomar JL, Mestres CA, Gherli T (2007). Heart fatty acid binding protein in the diagnosis of myocardial infarction: where do we stand today?. Cardiology.

[9] Kimura H, Fujii H, Suzuki S, Ono T, Arakawa M, Gejyo F (1999). Lipid-binding proteins in rat and human kidney. Kidney Int Suppl.

[10] Setsuta K, Seino Y, Kitahara Y, Arau M, Ohbayashi T, Takano T, Mizuno K (2008). Elevated levels of both cardiomyocyte membrane and myofibril damage markers predict adverse outcomes in patients with chronic heart failure. Circ J.

[11] Akbal E, Ozbek M, Gunes F, Akyurek O, Ureten K, Delibasi T (2009). Serum heart type fatty acid binding protein levels in metabolic syndrome. Endocrine.

[12] Haase-Fielitz A, Haase M, Bellomo R, Lambert G, Matalanis G, Story D, Doolan L, Buxton B, Gutteridge G, Luft FC, Schnuck W-H, Dragun D (2009). Decreased catecholamine degradation associates with shock and kidney injury after cardiac surgery. J Am Soc Nephrol.

[13] Yan X, Jia S, Meng X, Dong P, Jia M, Wan J, Hou X (2010). Acute kidney injury in adult postcardiotomy patients with extracorporeal membrane oxygenation: evaluation of the RIFLE classification and the Acute Kidney Injury Network criteria. Eur J Cardiothorac Surg.

[14] Li SY, Chen JY, Yang WC, Chuang CL (2011). Acute kidney injury network classification predicts in-hospital and long-term mortality in patients undergoing elective coronary artery bypass grafting surgery. Eur J Cardiothorac Surg.

[15] Olsson D, Sartipy U, Braunschweig F, Holzmann MJ (2013). Acute kidney injury following coronary artery bypass surgery and long-term risk of heart failure. Circ Heart Fail.

[16] Mehta RL, Kellum JA, Shah SV, Molitoris BA, Ronco C, Warnock DG, Levin A, Acute Kidney Injury Network (2007). Acute Kidney Injury Network: report of an initiative to improve outcomes in acute kidney injury. Crit Care.

[17] Haase M, Bellomo R, Devarajan P, Ma Q, Bennett MR, Mockel M, Matalanis G, Dragun D, Haase-Fielitz A (2009). Novel biomarkers early predict the severity of acute kidney injury after cardiac surgery in adults. Ann Thorac Surg.

[18] Gaudino M, Luciani N, Giungi S, Caradonna E, Nasso G, Schiavello R, Luciani G, Possati G (2005). Different profiles of patients who require dialysis after cardiac surgery. Ann Thorac Surg.

[19] Kuitunen A, Vento A, Suojaranta-Ylinen R, Pettila V (2006). Acute renal failure after cardiac surgery: evaluation of the RIFLE classification. Ann Thorac Surg.

[20] Haase M, Bellomo R, Matalanis G, Calzavacca P, Dragun D, Haase-Fielitz A (2009). A comparison of the RIFLE and Acute Kidney Injury Network classifications for cardiac surgery-associated acute kidney injury: a prospective cohort study. J Thorac Cardiovasc Surg.

[21] Katagiri D, Doi K, Honda K, Negishi K, Fujita T, Hisagi M, Ono M, Matsubara T, Yahagi N, Iwagami M, Ohtake T, Kobayashi S, Sugaya T, Noiri E (2012). Combination of two urinary biomarkers predicts acute kidney injury after adult cardiac surgery. Ann Thorac Surg.

[22] Arthur JM, Hill EG, Alge JL, Lewis EC, Neely BA, Janech MG, Tumlin JA, Chawla LS, Shaw AD (2014). Evaluation of 32 urine biomarkers to predict the progression of acute kidney injury after cardiac surgery. Kidney Int.

[23] Liu S, Che M, Xue S, Xie B, Zhu M, Lu R, Zhang W, Qian J, Yan Y (2013). Urinary L-FABP and its combination with urinary NGAL in early diagnosis of acute kidney injury after cardiac surgery in adult patients. Biomarkers.

[24] Peco-Antic A, Ivanisevic I, Vulicevic I, Kotur-Stevuljevic J, Ilic S, Ivanisevic J, Miljkovic M, Kocev N (2013). Biomarkers of acute kidney injury in pediatric cardiac surgery. Clin Biochem.

[25] Cabiati M, Caselli C, Caruso R, Prescimone T, Verde A, Botta L, Parodi O, Del Ry S, Giannessi D (2013). High peripheral levels of h-FABP are associated with poor prognosis in end-stage heart failure patients with mechanical circulatory support. Biomark Med.

[26] Pleym H, Tjomsland O, Asberg A, Lydersen S, Wahba A, Bjella L, Dale O, Stenseth R (2005). Effects of autotransfusion of mediastinal shed blood on biochemical markers of myocardial damage in coronary surgery. Acta Anaesthesiol Scand.

[27] The International Expert Committee (2009). International Expert Committee report on the role of the A1C assay in the diagnosis of diabetes. Diabetes Care.

[28] Aronson S, Fontes ML, Miao Y, Mangano DT, Investigators of the Multicenter Study of Perioperative Ischemia Research Group (2007). Risk index for perioperative renal dysfunction/failure: critical dependence on pulse pressure hypertension. Circulation.

[29] Nauta FL, Boertien WE, Bakker SJ, van Goor H, van Oeveren W, de Jong PE, Bilo H, Gansevoort RT (2011). Glomerular and tubular damage markers are elevated in patients with diabetes. Diabetes Care.

[30] Vellinga S, Verbrugghe W, De Paep R, Verpooten GA, Janssen van Doorn K (2012). Identification of modifiable risk factors for acute kidney injury after cardiac surgery. Neth J Med.

[31] Hong S, Youn Y-N, Yoo K-J (2010). Metabolic syndrome as a risk factor for postoperative kidney injury after off-pump coronary artery bypass surgery. Circ J.

[CR32] The pre-publication history for this paper can be accessed here:http://www.biomedcentral.com/1471-2261/14/117/prepub

